# Deciphering the puzzle: a case report of Tjalma syndrome (pseudo-pseudo Meigs' syndrome) with profoundly elevated CA-125 and pleural effusion

**DOI:** 10.3389/fimmu.2023.1277683

**Published:** 2023-12-13

**Authors:** Qing Tan, Li Feng Xu, Ting Yan, Cheng Hui Huang, Yi Tao, Wen Hui Huang, Shui Lian Yu

**Affiliations:** ^1^ Department of Rheumatology, Second Affiliated Hospital, Guangzhou Medical University, Guangzhou, Guangdong, China; ^2^ Department of Cardiology, Second Affiliated Hospital, Guangzhou Medical University, Guangzhou, Guangdong, China; ^3^ Department of Clinical Medicine, The Second Clinical School of Guangzhou Medical University, Guangzhou Medical University, Guangzhou, Guangdong, China

**Keywords:** pseudo-pseudo Meigs’ syndrome, Tjalma syndrome, pleural effusion, CA-125, systemic lupus erythematosus

## Abstract

Elevated CA-125 levels, polyserous effusions (such as pleural effusion, ascites, etc.) in young women with systemic lupus erythematosus (SLE) may signal pseudo-pseudo Meigs’ syndrome (PPMS), after excluding other causes. We describe a 32-year-old SLE patient with recurrent bilateral pleural effusions and unexplained hypercalcemia for 10 months. Extensive evaluations revealed no infections or tumors. Cytokine analysis showed elevated interleukin (IL) levels, especially IL-6 in pleural effusion. Treatment with immunosuppressive therapy resulted in reduced cancer antigen (CA) 125 levels and decreased effusion volume, demonstrating a positive response to intervention in this case of PPMS.

## 1 Introduction

Tjalma provided a clinical description of pseudo-pseudo Meigs’ syndrome (PPMS), which manifests as the presence of polyserous effusions (such as pleural effusion, ascites, etc.), and elevated levels of cancer antigen (CA) 125 (1). This condition occurs in individuals diagnosed with systemic lupus erythematosus (SLE), and is not associated with the presence of any benign or malignant ovarian tumors.

Pseudo-pseudo Meigs’ syndrome (PPMS) is a rare clinical entity. PPMS was first reported by Tjalma. In young women with systemic lupus erythematosus (SLE), the coexistence of ascites, pleural effusion, and elevated CA-125, unrelated to malignancy, should raise suspicion of PPMS when tuberculosis, infection, and thoracic or abdominal malignancies are ruled out. Concurrently, differentiation from pseudo-Meigs’ syndrome (PMS) and Meigs’ syndrome (MS) is crucial. MS typically presents as a triad involving benign ovarian tumors, thoracic and ascitic fluid accumulation, and rapid symptom resolution post-tumor resection, primarily observed in women aged 50-60 (2). Conversely, pseudo-Meigs’ syndrome stems from non-ovarian pelvic tumors (3). PPMS, with diverse clinical manifestations, often leads to misdiagnoses such as tuberculosis, hepatitis, or malignant tumors. Vigilance in differential diagnoses is essential to ensure timely and accurate treatment. This report details a case of a young female with SLE, exhibiting recurrent massive bilateral pleural effusions, elevated CA-125, and serum calcium, thus broadening the understanding of PPMS.

## 2 Case presentation

**Figure 1:**
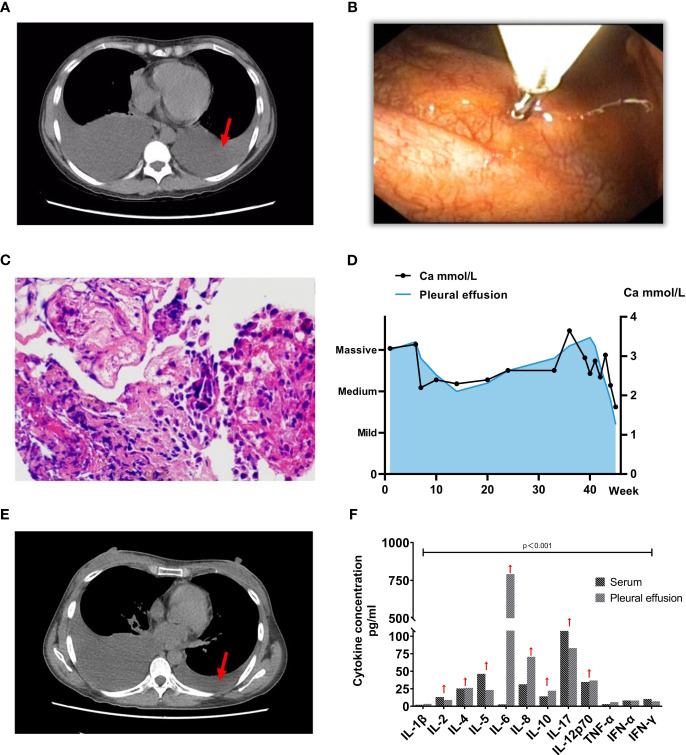
Clinical and Diagnostic Progression in Patient with PPMS and SLE. **(A)** Computed Tomography (CT) image depicting extensive pleural effusion before treatment (indicated by red arrows) (2022-10-17). **(B-C)** Subsequent to the histopathological examination of the parietal pleura, the pathology report indicates the presence of lymphocytes, histiocytes, and normal mesothelial cells, with no discernible tumor cells identified. The red arrow signifies values exceeding the normal range. **(D)** Graph showing the progression of serum calcium concentration, with the left vertical axis indicating pleural effusion volume and the horizontal axis representing time. Ultrasonography assessed the pleural effusion volume, with the first week of patient admission as the starting point. **(E)** Subsequent CT image illustrating a significant reduction in pleural effusion post-treatment (2022-11-16). **(F)** Cytokine concentration in both serum and pleural effusion before the initiation of immunosuppressive therapy.

A 32-year-old female patient presented with recurring bilateral pleural effusions, coinciding with her 8-year history of SLE. The patient’s SLE debut showcased widespread arthritis, predominantly targeting the knees. The physical examination (PE) demonstrated pain and restricted range of motion in the affected knee. Moreover, there was a reduction in breath sounds observed over the region encompassing both lung bases. After diagnosis, she received treatment comprising prednisone, hydroxychloroquine (HCQ), and methotrexate (MTX) for 3 years, which was subsequently discontinued upon achieving disease stability. During the medication hiatus, intermittent lower limb pain emerged, partially relieved by prednisone. Approximately ten months before hospitalization, she developed recurring dry cough and pronounced knee pain, prompting a computed tomography (CT) scan that revealed bilateral pleural effusions (**Figure 1A**). Thoracentesis and closed thoracic drainage were performed, yielding yellowish pleural effusion (2500 ml) with benign cytology.

Serological assessments revealed an ANA titer of 1/1000, anti-dsDNA antibodies at 52.14 IU/mmol (normal range: 0-12), weakly positive anti-Sm and anti-RNP antibodies, without proteinuria or hematuria. Treatment commenced with HCQ (200 mg daily) and methylprednisolone (MP) (16 mg daily). Notably, elevated serum calcium (3.3 mmol/L) and serum creatinine (146 μmol/L) levels were observed. A bone marrow biopsy disclosed no anomalies, while positron emission tomography-CT (PET-CT) revealed osteoporosis. Intramuscular salmon calcitonin was employed to mitigate hypercalcemia. Despite these measures, the patient encountered recurrent pleural effusions, leading to multiple hospitalizations within three months, marked by chest tightness and exertional dyspnea. Ultrasound examinations consistently revealed significant bilateral pleural effusions.

To ascertain a diagnosis, a right-sided medical thoracoscopy was performed, revealing interstitial lung inflammation with lymphoid follicular hyperplasia and pleural fibrous thickening (**Figures 1B, C**). Immunohistochemical staining of alveolar epithelium showed positivity for CD20 and CK, ruling out malignancy. Throughout five hospitalizations totaling 45 weeks, serum calcium levels remained elevated, with pleural fluid CA-125 consistently exceeding 1300 U/mL, peaking at 2767 U/mL (**Figure 1D**). Comprehensive evaluations excluded cardiac, thyroid-parathyroid, abdominal, and cranial abnormalities, while vaginal ultrasound indicated physiological findings. The patient fulfilled criteria for PPMS due to SLE-associated pleural effusion and elevated CA-125. Aggressive immunosuppressive therapy was initiated, accompanied by measures to manage hypercalcemia. Eighty milligrams per day of methylprednisolone therapy was given intravenously for 11 days, and then, the dosage was reduced to 40mg/day and continued intravenously for 3 days, subsequently, 1mg/kg/day prednisone acetate tablets were given orally as maintenance therapy, the prednisone acetate dosage was reduced by 2.5mg weekly and maintained at a dose of 10mg/day. Mycophenolate mofetil (MMF) is administered at a dosage of 750 mg twice per day. Hydroxychloroquine Sulfate Tablets were taken orally at 400 mg/day. One month after treatment with the hormone, MMF and Plaquenil, the clinical symptoms, and clinical test indexes of the patients were significantly improved (4, 5). The assessment of pleural effusion volume revealed a significant reduction (**Figure 1E**). Upon reevaluation during the November 2022 follow-up, the serum CA 125 level was measured at 42.10 U/ml, and urine protein quantitation yielded a result of 114 mg/24 h.

## 3 Discussion

PPMS, due to its rarity, is often prone to diagnostic challenges. In this case, the patient’s clinical presentation encompassed recurrent massive pleural effusions, bilateral knee pain, profound hypercalcemia, and immunological aberrations. Differential diagnosis meticulously excluded neoplastic, infectious, tuberculous, and rheumatologic etiologies for the pleural effusions. Despite multiple pleural fluid aspirations, the refractory nature of the effusions persisted. Intriguingly, histopathological analyses of lung and chest wall tissues yielded no evidence of malignancy or heterogeneous cell populations.

Diagnostic assessments, including vaginal ultrasound and PET-CT, effectively excluded ovarian tumors. Notably, serological and imaging evaluations dismissed malignancies, tuberculosis, and infections. The pleural effusion associated with SLE is typically bilateral and exudative (6). Consequently, the ensuing diagnostic framework pointed toward a lupus pleural effusion in the context of underlying SLE. This presumption was substantiated by the elevated CA-125 levels, which, following meticulous exclusion of diverse pathogenic conditions, prompted consideration of the possibility of PPMS. In women of childbearing age who have autoimmune diseases, the presence of endometriosis may lead to an elevation in the non-specific marker CA-125 (7). Nevertheless, a comprehensive assessment of the patient’s gynecological history reveals a regular menstrual cycle devoid of characteristic symptoms such as dysmenorrhea, dyspareunia, or chronic pelvic pain, thus diminishing the probability of endometriosis. Negative laboratory tests, including those for vaginal discharge, sex hormone levels, and human epididymal protein 4 (HE4), serve as valuable diagnostic tools for excluding alternative diseases. Etiologically, the raised CA-125 levels and exudative pleural effusion in the patient could potentially be associated with mesothelial cell activation triggered by SLE (4).

Mechanismly, T cells and their cytokines are well-known to have a key role in the pathogenicity of SLE. In our patient, serum cytokine analysis for this patient revealed heightened levels of IL-2, IL-4, IL-5, IL-10, IL-17, and IL-12p70 (**Figure 1F**). Notably, IFN-γ, IL-6, and IL-8 were also significantly elevated in the pleural effusion, with IL-6 reaching 792.23 pg/ml (normal: 7.0 pg/ml). This points toward the potential suitability of biologic treatments targeting inflammatory factors, including antibodies directed against the IL-23/IL-17 axis or Jak/Stat inhibitors (8). In the context of most PPMS patients, the administration of immunosuppressants and corticosteroids is the standard approach (4, 5). The attainment of remission has been realized through the strategic utilization of MMF, azathioprine, cyclophosphamide, and rituximab (9). Additional research is required to investigate the most advantageous moment for commencing treatment. It’s worth noting that hypocalcemia is more prevalent among individuals with lupus. Factors contributing to this phenomenon include patients’ tendency to avoid ultraviolet light, the potential for renal or gastrointestinal complications to hinder calcium absorption, and the potential for long-term hormone therapy to induce calcium loss, among other factors (10).

Significantly, our patient experienced hypercalcemia and impaired bone metabolism. In the complex context of SLE, hypercalcemia may arise from diverse mechanisms, including antibodies against parathyroid hormone-related peptides or stimulating receptors, heightened osteoclast activity leading to bone resorption, and pro-inflammatory cytokine disruption of bone turnover during active SLE phases (10). Hypercalcemia could potentially serve as a marker of SLE activity, correlating with autoantibody production and pro-inflammatory factors. It might even manifest as an initial sign of SLE (11). These perspectives warrant consideration as the underlying mechanism of hypercalcemia in PPMS patients.

## Data availability statement

The original contributions presented in the study are included in the article/supplementary material. Further inquiries can be directed to the corresponding author.

## Ethics statement

Ethical review and approval were not required for the study on human participants following the local legislation and institutional requirements. Written informed consent was obtained from the patient for the publication of this case report.

## Author contributions

QT: Writing – original draft. LX: Writing – review & editing. TY: Writing – review & editing. CH: Writing – review & editing. YT: Writing – review & editing. WH: Writing – review & editing. SY: Writing – review & editing.
